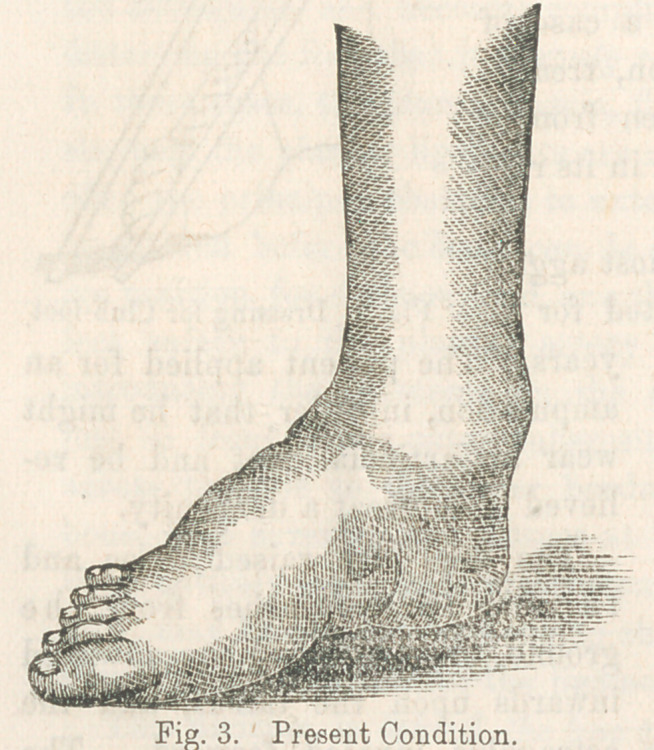# Deformities of the Feet

**Published:** 1867-08

**Authors:** Julien S. Sherman

**Affiliations:** Chicago


ARTICLE XXXII.

### DEFORMITIES OF THE FEET.

BY JULIEN S. SHERMAN, M.D., Chicago.

The feet are more frequently the seat of deformity, either congenital or acquired, than any other members of the body, and the important part they perform in the act of walking, renders all the forms of talipes a serious impediment to locomotion. With the exception of spinal curvatures, they require more perseverance and continued effort for their correction than deformities of other localities. Improvements in the principles of treatment and manner of dressing have added greatly to the success in relieving them, so that at present there are but very few cases, even where the distortion is great, that will not yield to persistent and well-directed efforts, so that at least a useful if not a perfect member may be obtained.

These deformities are not generally rapidly produced, neither are they quickly cured; but their removal requires a period reaching through weeks and, often, months.

The causes which produce them are as numerous as the various forms under which they are seen. One, almost constant, factor in their pathology is *unequal muscular action.* Paralysis frequently results in these deformities. Inflammation of the ankle, by causing reflex contraction of the muscles in its vicinity, often produces talipes equinus, as the muscles of the calf are stronger than the extensors, they draw the heel up and produce the deformity. These, or other causes, which produce unequal shortening or contraction of muscles and ligaments, terminate in malposition of the foot.

Restoration is prevented by the resistance of these shortened tissues, and may be overcome in two ways:—by extension long continued, and by tenotomy. Some orthopædists have discarded operative measures entirely, claiming that the contracted tissues may all be lengthened by sufficient extension. This is true in many cases; but there are others, where the deformity is so great and of so long-standing that tenotomy offers the only hope of restoring the parts. A careful selection of cases should be made for the two methods of treatment, in order that the division of tendons may be as limited as possible. Properly adapted apparatus is necessary after the operation to complete the cure.

### DIVISION OF THE FLEXOR BREVIS DIGITORUM MUSCLE, AND PLANTAR FASCIA.

There is a rare deformity of the foot, in which the tarso-metatarsal articulation and the phalanges are the parts principally involved, the bones being so far drawn from their proper position that they point backward, and the patient walks upon the heads and dorsal aspect of the phalanges. If the limb is much used, the deformity increases to partial displacement of the astragalus, and becomes complicated with talipes varus, so distorting the foot that it *scarcely resembles the human in form.* In these cases, the plantar fascia, flexor brevis digitorum muscle, and the plantar ligaments are most firmly contracted, and offer the principal obstacles to extension. These tissues must be divided before the *bones can be extended.* The most effective position for division, and one that is approached with perfect safety to the plantar artery and nerve, is reached by introducing the tenotome on the outer side of the os calcis, just in front of the outer tuberosity, and carrying it directly across the sole to the inner border, and then cutting to the bone, thus severing the tissues at their origin and separating them entirely from their attachments. The long plantar ligament may be divided by keeping the knife still in contact, and carrying it forward until the ligament is separated.

This division is *behind the line of crossing of the artery and nerve*, hence they escape injury, and by it the phalanges are liberated and can be brought forward sufficiently to admit of suitable dressings for extension. An effective and simple apparatus for this purpose is represented by Fig. 1. It consists of a piece of sole leather, soaked until soft and pliable, and then moulded to the foot and laced at the side. A band of sheet steel is attached to the bottom and extends as a rod beyond the toes, at the end of which buckles are fastened. Adhesive straps are applied to the front and side of the leg, the lower ends of which terminate in elastic webbing, which is carried forward and secured in the buckles at the end of the steel rod. By these means the points of purchase on the leg and foot are obtained and the latter may then be drawn strongly in any desired direction, and the stretch continued until the contracted tissues have yielded, and the foot assumed the normal position.

Fig. 1. Dressing for Club-foot.

Fig. 2, is a sketch of a case of the previous description, treated last spring. It is taken from a cast, and is very accurate in its representation.

This case was of the most aggravated kind, and had existed for 22 years. The patient applied for an amputation, in order that he might wear an artificial foot and be relieved of so great a deformity.

Fig. 2.

The heel was raised three and three-quarter inches from the ground, the metatarsal bones twisted inwards upon the tarsus, and the astragalus luxated forward. The toes strongly extended, so that the dorsal surface of the fourth and fifth were in contact with the corresponding surfaces of the metatarsal bones. In standing, the point of contact with the ground was thrown back of the ankle-joint. The deformity was complicated with talipes varus.

It was necessary to make a complete division of the flexor brevis digitorum muscle, and plantar fascia, which was accomplished in the manner previously described. The tendo Achillis and tibialis posticus were also divided. The foot was then placed in the dressing represented by Fig. 1, and, subsequently, another apparatus provided, which, by means of a ratchet and cog, turned the sole of the foot outward, at the same time that the toes were elevated and the heel depressed. During the treatment, union of the tendo Achillis was so rapid that it required division three times, before the heel was brought into position. The case was under treatment three months, which, considering the age of the deformity and resistance of the tissues, was a rapid result.

Fig. 3. Present Condition.

Fig. 3, is taken from a photograph, and represents the present condition. The result is much better than was expected. The whole sole is in perfect contact with the ground, and the foot points out fully as much as its fellow. It is somewhat smaller than the other, from non-development, but otherwise is a strong and useful member. The muscles of the calf will increase in size and strength as the foot is used.

Deformities of the feet should never be neglected, but receive prompt attention at the earliest period possible, as the tissues become stronger and more contracted as the case advances in age. In very young children, caution should be used in applying apparatus, that ulceration of the skin is not produced. For such cases, the adhesive strap and elastic webbing form the best dressing. By these means, most cases in children, if taken early, can be cured without any operative measures.

## Figures and Tables

**Fig. 1. f1:**
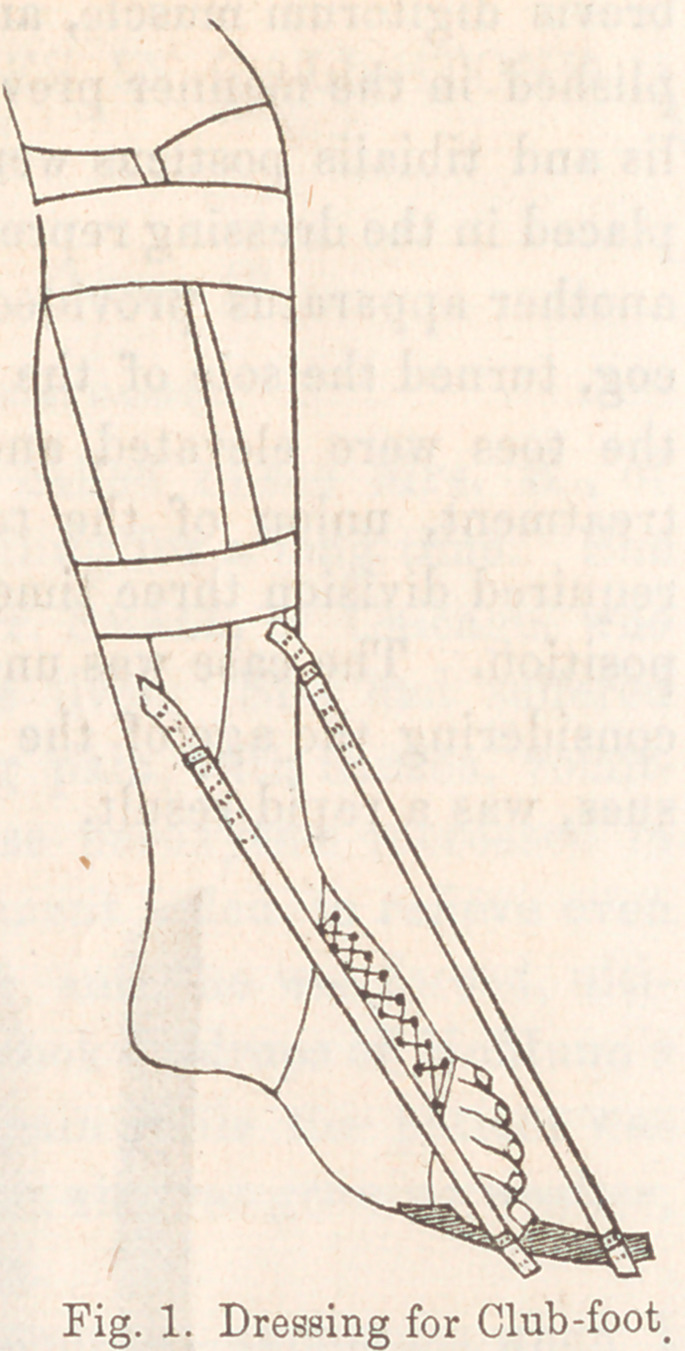


**Fig. 2. f2:**
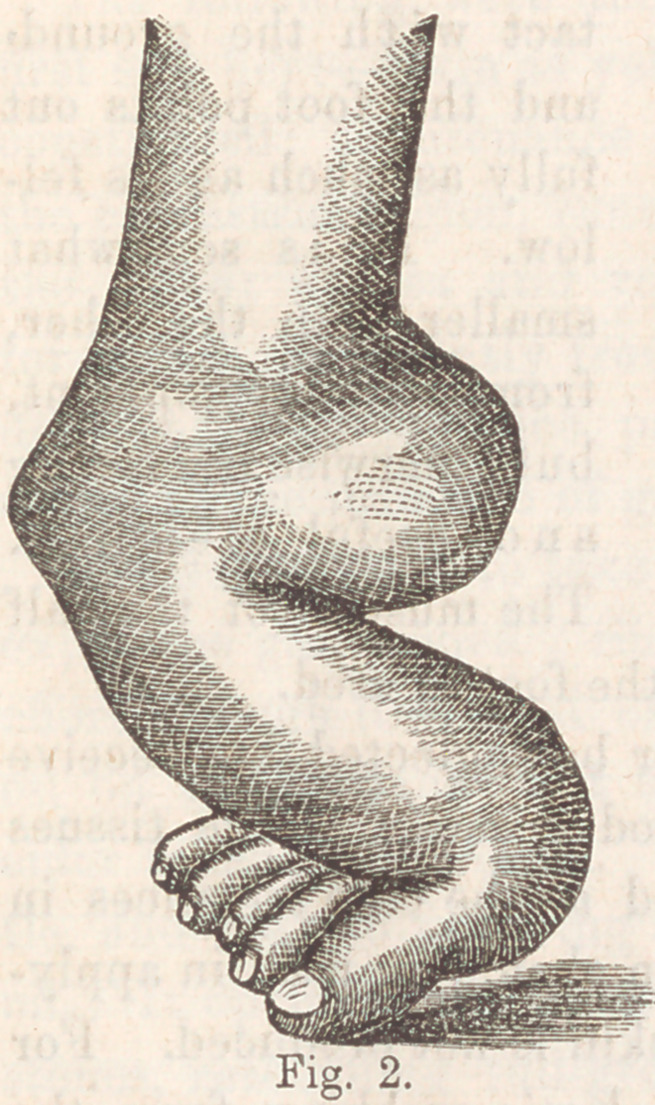


**Fig. 3. f3:**